# Oncogenic KRAS promotes malignant brain tumors in zebrafish

**DOI:** 10.1186/s12943-015-0288-2

**Published:** 2015-02-03

**Authors:** Bensheng Ju, Wenbiao Chen, Brent A Orr, Jan M Spitsbergen, Sujuan Jia, Christopher J Eden, Hannah E Henson, Michael R Taylor

**Affiliations:** Department of Chemical Biology & Therapeutics, St. Jude Children’s Research Hospital, Memphis, TN 38105 USA; Department of Molecular Physiology & Biophysics, Vanderbilt University School of Medicine, Nashville, TN 37232 USA; Department of Pathology, St Jude Children’s Research Hospital, Memphis, TN 38105 USA; Fish Disease Research Group, Department of Microbiology, Oregon State University, Corvallis, OR 97331 USA; Department of Developmental Neurobiology, St. Jude Children’s Research Hospital, Memphis, TN 38105 USA; Integrated Program in Biomedical Sciences, University of Tennessee Health Science Center, Memphis, TN 38163 USA; Current address: Pharmaceutical Sciences Division, School of Pharmacy, University of Wisconsin-Madison, Madison, WI 53705 USA

**Keywords:** Zebrafish, Oncogenic KRAS (KRAS^G12V^), *krt5*, *gfap*, Brain tumors, Drug screening

## Abstract

**Background:**

Zebrafish have been used as a vertebrate model to study human cancers such as melanoma, rhabdomyosarcoma, liver cancer, and leukemia as well as for high-throughput screening of small molecules of therapeutic value. However, they are just emerging as a model for human brain tumors, which are among the most devastating and difficult to treat. In this study, we evaluated zebrafish as a brain tumor model by overexpressing a human version of oncogenic KRAS (KRAS^G12V^).

**Methods:**

Using zebrafish *cytokeratin 5* (*krt5*) and *glial fibrillary acidic protein* (*gfap*) gene promoters, we activated Ras signaling in the zebrafish central nervous system (CNS) through transient and stable transgenic overexpression. Immunohistochemical analyses were performed to identify activated pathways in the resulting brain tumors. The effects of the MEK inhibitor U0126 on oncogenic KRAS were evaluated.

**Results:**

We demonstrated that transient transgenic expression of KRAS^G12V^ in putative neural stem and/or progenitor cells induced brain tumorigenesis. When expressed under the control of the *krt5* gene promoter, KRAS^G12V^ induced brain tumors in ventricular zones (VZ) at low frequency. The majority of other tumors were composed mostly of spindle and epithelioid cells, reminiscent of malignant peripheral nerve sheath tumors (MPNSTs). In contrast, when expressed under the control of the *gfap* gene promoter, KRAS^G12V^ induced brain tumors in both VZs and brain parenchyma at higher frequency. Immunohistochemical analyses indicated prominent activation of the canonical RAS-RAF-ERK pathway, variable activation of the mTOR pathway, but no activation of the PI3K-AKT pathway. In a *krt5*-derived stable and inducible transgenic line, expression of oncogenic KRAS resulted in skin hyperplasia, and the MEK inhibitor U0126 effectively suppressed this pro-proliferative effects. In a *gfap*-derived stable and inducible line, expression of oncogenic KRAS led to significantly increased mitotic index in the spinal cord.

**Conclusions:**

Our studies demonstrate that zebrafish could be explored to study cellular origins and molecular mechanisms of brain tumorigenesis and could also be used as a platform for studying human oncogene function and for discovering oncogenic RAS inhibitors.

**Electronic supplementary material:**

The online version of this article (doi:10.1186/s12943-015-0288-2) contains supplementary material, which is available to authorized users.

## Background

Brain tumors account for approximately 90% of all CNS tumors. According to the National Cancer Institute, there have been 23,130 new cases of brain tumors and other nervous system cancers in 2013 with 14,080 deaths occurring in the United States alone (http://www.cancer.gov/cancertopics/types/brain). The inaccessibility of some brain tumors to surgery, limitations in drug delivery due to presence of the brain–blood barrier (BBB), and the malignant nature of some tumor types make brain tumors especially hard to treat. For example, children with diffuse intrinsic pontine glioma (DIPG) and adults with glioblastoma multiforme (GBM) have a dismal prognosis, and no significant progress has been made over the past several decades to improve survival rates [1,2].

Although Ras mutations are uncommon in human brain tumors, activation of the canonical Ras pathway through receptor tyrosine kinases is a common mechanism for human glioblastoma development [3]. By activating these pathways, several mouse glioma models have been generated, primarily through overexpression of oncogenic KRAS [4-7].

Zebrafish have emerged as an important model to study human cancers and to understand tumorigenic mechanisms [8] and the Ras pathway activation had been successfully used to induce melanoma [9], pancreatic cancer [10], embryonal rhabdomyosarcoma [11], and liver cancer [12]. To evaluate whether zebrafish could be used as a brain tumor model, we expressed the human version of KRAS^G12V^ driven by the zebrafish *krt5* promoter that we recently identified [13] and also by the well-characterized *gfap* gene promoter [14]. We demonstrated that zebrafish develop high-grade brain and other cranial tumors with variable penetrance in transient transgenic fish, which was promoter dependent. We also showed that U0126, a MAP kinase (MEK) inhibitor, could suppress the pro-proliferative effects of oncogenic KRAS, suggesting zebrafish could potentially be used as *in vivo* models to screening for Ras inhibitors that may prove to be of therapeutic value to a variety of human cancers with activated RAS signaling, including certain types of brain tumors.

## Results

### Zebrafish *krt5* promoter drives transgenic expression in the brain

**Figure 1:**
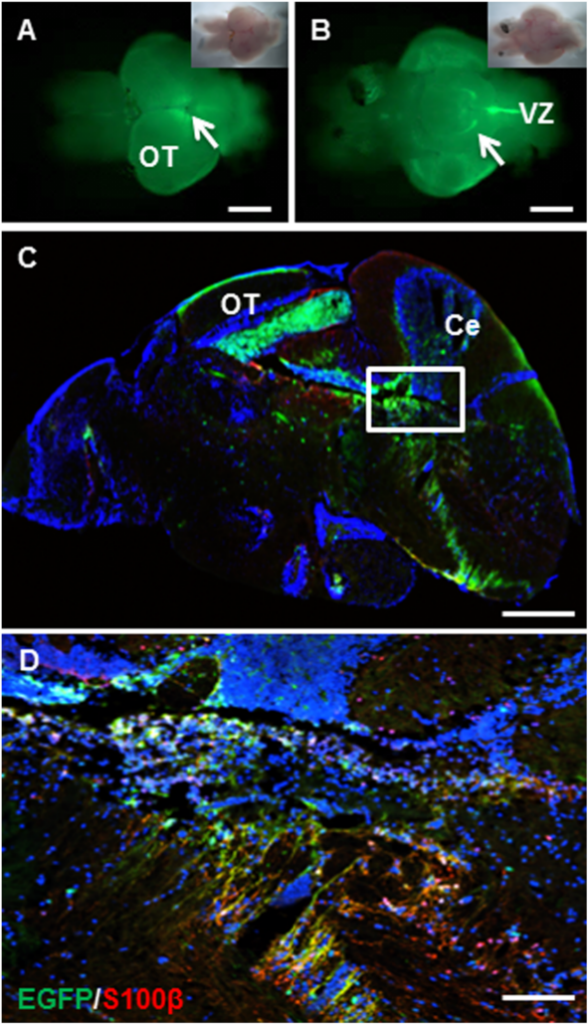
**Expression pattern of** ***Tg(krt5:EGFP)*** **in the adult zebrafish brain. (A)** Dorsal view (anterior to the left) of a 6-month-old transgenic fish brain showing EGFP expression in the ventricular zone (VZ) of the midbrain and hindbrain (arrow). Inset shows the bright-field view of the same brain. **(B)** Ventral view (anterior to the left) of the same brain as in **(A)** showing EGFP expression in the lobus inferior (LI) of the hypothalamus (arrow) and VZ of the hindbrain. **(C)** A sagittal section through the adult brain showing EGFP expression in the optic tectum (OT), cerebellum (Ce), and VZ. **(D)** Enlarged view of framed area in **(C)** showing overlapping EGFP expression and antibody staining for the radial glia marker S100β (red). Scale bars, 200 μm for A-C; 20 μm for D.

Zebrafish *krt5* gene shares conserved synteny with its mammalian counterparts [15]. Based on *in situ* hybridization, *krt5* is expressed in skin epithelial cells, neurons and glial cells of the brain and spinal cord, and chondrocytes of the skull [16]. Using a transgene consists of a 4.9 kb fragment of the *krt5* gene promoter and EGFP reporter, we developed two stable transgenic lines, *Tg(−4.9krt5:EGFP)*, both showing EGFP expression that faithfully recapitulates the endogenous *krt5* expression pattern in skin epithelial cells (Additional file 1: Figure S1A), radial glial cells (Additional file 1: Figure S1B), and chondrocytes (Additional file 1: Figure S1C). Unlike the well-characterized zebrafish *gfap* and *nestin* promoters that drive strong expression during early stages of brain development [14,17], our *Tg(−4.9krt5:EGFP)* lines showed EGFP expression in only a subset of neural cells (Additional file 1: Figure S1B, S1C). In adults, EGFP expression was found in the optic tectum (OT) and the dorsal side of the midbrain and hindbrain boundary (Figure 1A). On the ventral side of the brain, EGFP was prominent in the ventral regions lining the ventricular zones (VZ) of the midbrain and hindbrain and in the lobus inferior (LI) of the hypothalamus in a pattern similar to the zebrafish *midkine* gene [18] (Figure 1B). Sagittal sections of the adult brain confirmed expression on the surface of the OT and in the VZs (Figure 1C). EGFP-positive cells in the brain VZs were morphologically similar to radial glial cells and their expression patterns partially overlap with the radial glia marker S100β (Figure 1D).

### Overexpression of *KRAS*^*G12V*^ in *krt5*-expressing cells leads to malignant tumors of cranial cavity

**Figure 2:**
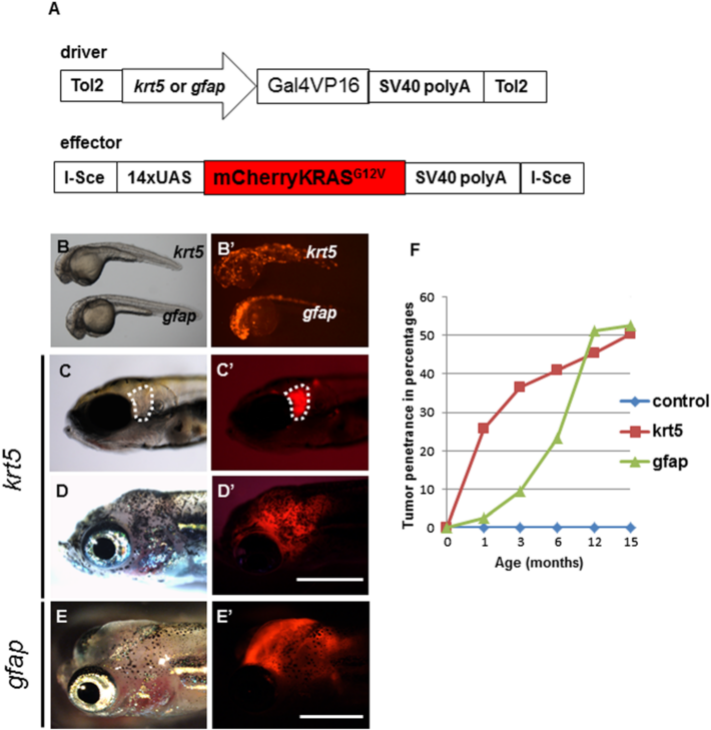
**Transient expression of oncogenic KRAS results in tumorigenesis in the brain region. (A)** Graphic representation of driver and effector DNA constructs used for co-injection into zebrafish eggs. **(B, B’)** The *krt5* gene promoter directed prominent transgenic expression in skin epithelial cells and other cell types, whereas the *gfap* promoter directed expression primarily in the CNS. **(C, C’)** A 9-day old *krt5*-transgenic fish showing focal expression of the oncogenic mCherry-KRAS in the head (outlined in white). **(D, D’)** A *krt5*-transgenic fish at 2 months of age showed gross tumor infiltration within the head. **(E, E’)** A *gfap*-transgenic fish at 2 months of age showed similar tumor infiltration in the head. **(F)** Both *krt5*- and *gfap*-transgenic fish developed tumors within the head region at approximately 50% penetrance by 15 months of age. Scale bars, 0.5 cm.

We used a co-injection strategy to deliver Tol2-based *Tg*(*krt5:Gal4VP16)* and I-SceI meganuclease*-*based *Tg(UAS:mCherry-KRAS*^*G12V*^*)* constructs into individual single-cell embryos (Figure 2A). Each embryo received approximately 20 pg of the combined plasmid DNA, as higher \doses caused severe abnormalities and high mortality. When transiently expressed in zebrafish embryos, *krt5* drove mCherry expression prominently in skin epithelial cells and other cell types, including cells in the brain when observed at 24 hours post-fertilization (hpf) (Figure 2B,B’). Despite this broad expression, we only observed gross tumor formation in the head region. The earliest sign of tumorigenesis was observed in a 9-day-old larva that showed a tumor mass between the eye and the ear (Figure 2C, C’). By approximately 1 month of age, 25.8% (*n* = 17/66) of fish began to develop tumors. By 2 months of age, tumors in some fish were visible to the naked eye (Figure 2D) and emitted strong mCherry fluorescence when observed under a fluorescence microscope (Figure 2D’). At 1 year of age, tumors were seen in approximately 50% of transient transgenic fish (Figure 2F).

**Figure 3:**
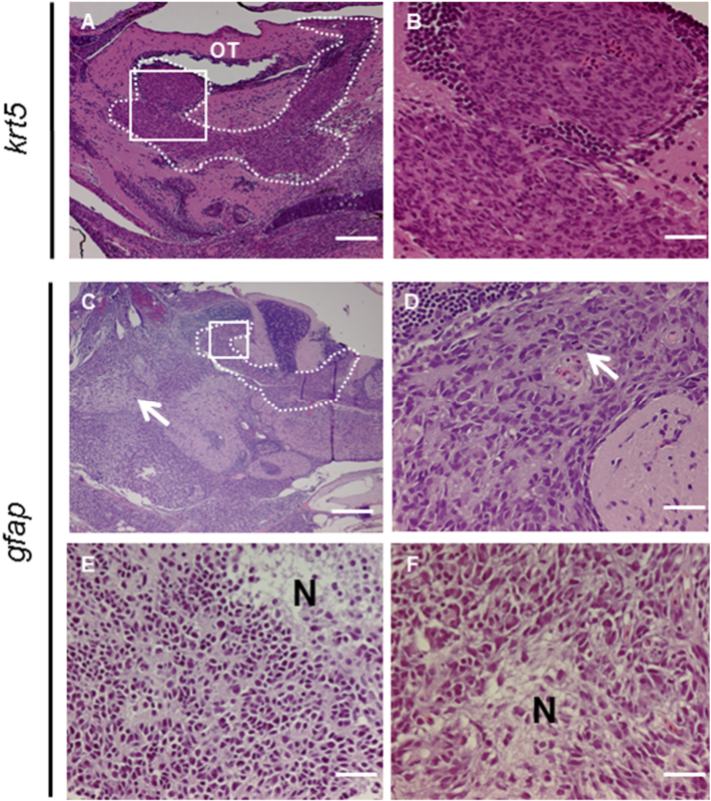
**Histological and immunohistological analyses of tumors from** ***krt5*** **and** ***gfap*** **transgenic fish. (A)** H&E staining of a sagittal section from a 6-month-old *krt5*-derived tumor showing tumor cells originating from and infiltrating the VZ of the optic tectum (OT), as highlighted by the dotted line. **(B)** Enlarged view of the white frame in (A) demonstrating infiltrating malignant cells with moderate pleomorphism, typical of high-grade astrocytoma. **(C)** A 6-month-old *gfap*-transgenic fish brain showing tumor development in both the VZ (broken line) and the brain parenchyma (arrow). *gfap*:*KRAS* ^*G12V*^-derived brain tumors exhibited phenotypes consistent with malignant glioma including frequent mitotic figures **(D)** and focal necrosis **(E, F)**. N, necrosis. Scale bars, 100 μm for **A**; 20 μm for **B**, **D**, **E** and **F**; 40 μm for **C**.

Hematoxylin and eosin (H&E) staining of paraffin sections from 15 tumor-bearing fish revealed 3 cases (20%, Table 1) with tumors originating from the brain region. These tumors infiltrated the VZ and exhibited histopathological features of glial tumors (Figure 3A-B). Despite this striking similarity in morphology, the 3 brain tumors did not show prominent expression of the glial markers of GFAP and S100β (Additional file 1: Figure S2A-B). The other 12 tumors exhibited biphasic cell morphology, with spindle cells mingling with epithelioid cells (Additional file 1: Figure S3A-D). These tumors usually possessed abundant mitotic figures (Additional file 1: Figure S3B) and were often locally invasive and spread to adjacent soft tissue (Additional file 1: Figure S3A) or gills (Additional file 1: Figure S3C). The spindle cell morphology and invasive nature of the tumors were reminiscent of zebrafish MPNSTs [19].

**Table 1:** **KRAS-induced tumors in zebrafish and immunohistochemical characterization of brain tumors**

**Promoters**	***krt5***	***gfap***
Tumors analyzed	15	10
Number of brain tumors	3 (20%)	6 (60%)
MPNST-like	12 (80%)	1 (10%)
Others	0 (0%)	3 (30%)
pERK1/2	3/3 (+++)	6/6 (+++)
pAKT (Ser473)	0/3	0/6
p4E-BP1	3/3 (++)	6/6 (++)
pS6RP	3/3 (+)	6/6 (5+; 1+++)

Immunofluorescence levels: +++, strong; ++ intermediate; + low and sporadic.

### Overexpression of *KRAS*^*G12V*^ in *gfap*-expressing cells leads to parenchymal brain tumors

The low frequency of brain tumors from the *krt5* gene promoter prompted us to test whether the well-characterized, broadly-expressing promoter of zebrafish radial glia gene *gfap* could induce higher tumor incidence [14]. As expected, the *gfap* promoter directed transgenic expression throughout the CNS (Figure 2B,B’). Despite strong expression of the oncogene, only about 50% (*n* = 22/43) of transgenic fish developed tumors in the head region at 1 year of age (Figure 2E, E’), making the overall tumor penetrance of transient *Tg(gfap:Gal4VP16; UAS:mCherry-KRAS*^***G12V***^***)*** transgenic fish similar to that of tumors from *Tg(krt5:Gal4VP16; UAS:mCherry-KRAS*^***G12V***^***)*** (Figure 2F).

Analysis of H&E-stained paraffin sections revealed that 6 of 10 *gfap*-derived tumors were brain tumors (Table 1). In affected fish, hypercellularity was observed throughout the brain (Figure 3C), consistent with the strong and ubiquitous nature of the *gfap* promoter. The neoplasms consisted of moderately pleomorphic cells with an infiltrative growth pattern. Mitotic activity was abundant (Figure 3D) and necrosis was encountered (Figure 3E, 3 F) in selected tumors, yet microvascular infiltration was largely absent. These overall characteristics were consistent with that seen in human high grade astrocytomas. Despite the presumed radial glial cell of origin and glial histomorphology, none of the tumors showed notable GFAP or S100β expression (Table 1). Among 4 peripheral tumors, 1 exhibited MPNST-like spindle cell morphology (Additional file 1: Figure S4A-B), while the other 3 consisted of undifferentiated neoplasms that were difficult to classify (Additional file 1: Figure S4C-D).

### Activation of Ras and mTOR pathways in brain tumors

**Figure 4:**
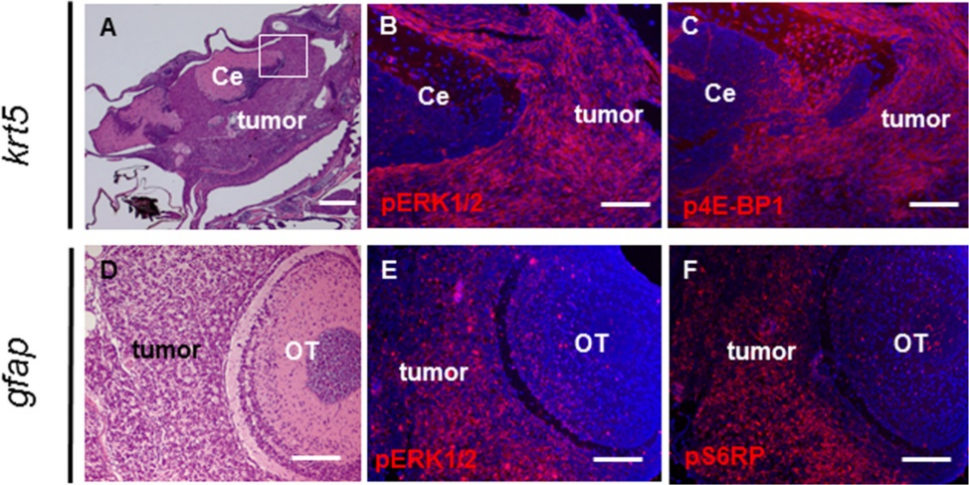
**Activation of the Ras and mTOR pathways in brain tumors. (A)** A 6-month-old *krt5*-derived brain tumor infiltrating both the ventral brain and the VZ. Tumor cells exhibited prominent expression of phospho-ERK1/2 **(B)** and phospho-4E-BP1 **(C)**. **(B)** and **(C)** show immunofluorescence staining of the white framed region in **(A)**. Note the relatively normal cerebellum exhibited much less staining for the two antibodies. **(D)** A 12-month-old *gfap*-derived brain tumor showed tumor cell infiltration of the VZ surrounding the OT. Tumor cells exhibited prominent expression of pERK1/2 **(E)** and pS6RP **(F)**. Note that paraffin sections were used for immunofluorescence. Ce, cerebellum; OT, Optic tectum. Scale bars, 200 μm for **A**; 40 μm for **B**, **C**, **D**, and **E**.

In mouse models of oncogenic Kras-induced glioma, tumor cells have increased expression of both phosphorylated ERK and phosphorylated AKT [7,20], indicating activation of the canonical Ras and PI3K-AKT pathways. To determine whether these pathways were simultaneously activated in zebrafish tumors, we performed extensive immunohistochemical analysis on tumor samples with antibodies against the downstream targets of these pathways. We found that both *krt5-* and *gfap*-derived brain tumors showed prominent phospho-ERK1/2 expression (Table 1 and Figure 4B,E). Next, we analyzed the PI3K-AKT pathway activation in brain tumors using a previously validated anti-phospho-AKT (Ser473) antibody [21] (Additional file 1: Figure S2C). None of the 9 brain tumors examined showed positive staining, suggesting that the PI3K-AKT pathway was not activated (Additional file 1: Figure S2D). However, all tumor samples were positive for phospho-4E-binding protein 1 (p4E-BP1; Figure 4C) and phospho-ribosomal protein S6 (pS6RP; Figure 4F), which are targets of the mammalian target of rapamycin (mTOR) pathway [22]. Unlike the prominent phospho-ERK1/2 staining, p4E-BP1 and pS6RP showed variability among tumor samples (Table 1). These results suggested that activation of the canonical Ras pathway and the mTOR pathway play key roles in KRAS- induced brain tumors in zebrafish.

### Generation of inducible transgenic lines for chemical screening

**Figure 5:**
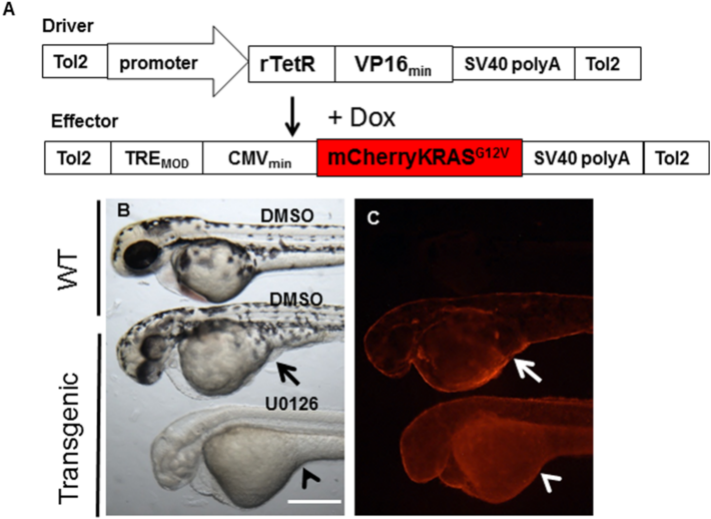
**MEK inhibitor U0126 suppresses proliferative effect of oncogenic KRAS in skin epithelial cells. (A)** Graphic representation of driver and effector DNA constructs for generation of Dox-inducible stable transgenic lines. **(B, C)** In an inducible Tg(*krt5:rtTA:mCherryKRAS* ^*G12V*^) transgenic line, 48hpf larvae treated with 10 μg/ml Dox showed skin hyperplasia, most noticeably under the yolk sac (arrows). 50 μM U0126 treatment completely eliminated the skin hyperplasia (arrow heads). Scale bar, 250 μm.

Since we did not expect that stable transgenic fish overexpressing the oncogenic KRAS to survive to adulthood, we further generated transgenic lines that allow Doxycycline (Dox) inducible expression of oncogenic KRAS in *krt5*- and *gfap*-expressing cells using the TetOn system (Figure 5A). In the presence of 10 μg/ml of Doxycycline starting from early gastrula stage, *Tg*(*krt5:rtTA;TRE:mCherry-KRAS*^***G12V***^) embryos showed weak mCherry expression in skin epidermal cells and skin hyperplasia was visible at 24hpf. Skin hyperplasia became more conspicuous at 48hpf, especially at the ventral yolk sac (Figure 5B). This hyperplasia could be effectively eliminated by treating embryos simultaneously with 50 μM of U0126 (Figure 5B-C; Additional file 1: Figure S5A-B, n = 24), a strong MEK inhibitor that blocks ERK phosphorylation when applied at the early gastrula stage of zebrafish embryonic development [23].

**Figure 6:**
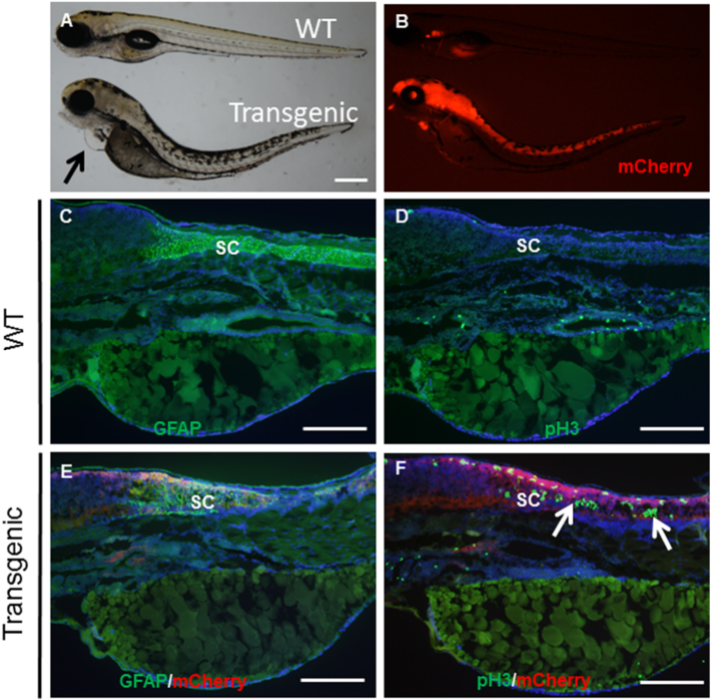
**Oncogenic KRAS expression in CNS increases mitotic activity in the spinal cords. (A, B)** 120hpf larvae from an inducible Tg(*gfap:rtTA:mCherryKRAS* ^*G12V*^) line showed heart edema (arrow), body curvature, and hyperpigmentation of the trunk. At 72hpf, wild-type larvae showed prominent expression of GFAP in the spinal cord **(C)**, and few cells were undergoing mitosis **(D)**. Expression of oncogenic KRAS disrupted the normal GFAP expression pattern in the spinal cord **(E)**, and significantly increased the number of cells undergoing mitosis (**F**, arrows). SC, spinal cord. Scale bars, 250 μm.

We also generated a *Tg(gfap:rtTA;TRE:mCherry-KRAS*^***G12V***^*)* line. Embryos from this line showed strong mCherry expression throughout the CNS at 24hpf in the presence of Dox. At 72hpf, transgenic larvae exhibited heart edema, body curvature and hyperpigmentation of the trunk, and these phenotypes were more obvious at 120hpf (Figure 6A-B). Sagittal sectioning of 72hpf larvae revealed that expression of oncogenic KRAS in CNS resulted in abnormal expression of GFAP (Figure 6C,E), and significantly increased mitotic figures in the spinal cords as indicated by immunostaining for phosphorylated Histone 3 (pH3) (Figure 6D,E). The mitotic figures in a 10 μm section spanning a region above the yolk sac increased from 1 ± 0.71 (n = 4) to 26 ± 9.80 (n = 6) (unpaired student’s *t*-test, p < 0.001). Surprisingly, increased mitosis was only observed in the region corresponding to strong endogenous GFAP expression, but not in brain regions showing negligible GFAP expression (Figure 6E-F). We attempted to evaluate whether U0126 treatment could reduce mitotic activities. Though the effective dosage of 100 μM applied at the early gastrula stage could reduce the mCherry levels in CNS (Additional file 1: Figure S6C-D), it also caused severe developmental defects by 48hpf. A much lower dose of 5 μM of U0126 was much less toxic, but did not significantly reduce the mitotic index (Data not shown).

## Discussion

In this study, we report that human oncogenic KRAS driven by the zebrafish *krt5* or *gfap* promoter induces malignant tumors of the nervous system. We demonstrated that the canonical Ras and mTOR signaling pathways were activated in tumors driven by both promoters. Furthermore, we generated inducible transgenic lines that exhibited epithelial hyperplasia in skin and increased mitotic activities in the spinal cords, respectively. We further showed that the MEK inhibitor U0126 suppressed the pro-proliferative effects of oncogenic KRAS in early embryonic development.

### Tumor types generated by oncogenic KRAS

The combined features of diverse tumor cell morphology, high cellularity, presence of mitotic figures and regional necrosis indicate that the onogenic KRAS-induced tumors are most likely malignant gliomas. Yet, the absence of GFAP and S100β expression in the adult brain tumors is intriguing. Both human and mouse models of glioma can lose GFAP expression with tumor progression either through epigenetic regulation [24] or tumor cell dedifferentiation [25]. Oncogenic KRas has also been found to cause defects in terminal differentiation of stem or progenitor cells in mouse colon [26] and zebrafish pancreatic [10] cancer models, so it is possible that GFAP expression was lost early during oncogenic KRAS-driven brain tumorigenesis. We also analyzed early stages of brain tumors at 18 days post-fertilization (dpf) and 30 dpf respectively, but found no obvious GFAP expression associated with tumorigenesis (Data not shown). Although oncogenic KRAS-induced brain tumors reported in this study resembled astrocytoma morphologically, we cannot exclude the possibility that they are a type of undifferentiated brain tumors, such as primitive neuroectodermal tumors (PNET) which often do not express GFAP [27].

The other KRAS-derived tumors with spindle cell morphology were compatible with human MPNSTs, which are aggressive nerve sheath tumors associated with activation of the RAS pathway. While MPNSTs generally arise in the setting of inactivating mutations in the neurofibromatosis 1 (*NF1*) gene [28], others have demonstrated that Kras is an important determinant of survival in MPNST [29]. Zebrafish carrying mutations in *tp53* [19] and several ribosomal protein genes [30] spontaneously develop MPNSTs in abdominal cavities or peri-ocular regions. Tumors observed in our transgenic fish represent another type of MPNST that could have a neural crest origin as the *krt5* promoter drives transgenic expression along this lineage (Additional file 1: Figure S1C).

### Cell origin and mechanism of brain tumorigenesis

Brain tumors can originate from specific brain regions and from different cell types [31]. There is heterogeneity in progenitor cell subtypes in the ventricular zone of the zebrafish adult telencephalon and other brain regions [32]. From the observation of a limited number of brain tumors, it appeared that *krt5*-derived tumors mostly originated from VZ, while *gfap*-derived tumors may have originated from both the VZ and brain parenchyma. These brain tumors exhibited different histological characteristics, supporting the concept that tumor cell origin affects tumor cell phenotypes [33].

The Ras and PI3K-AKT pathways are interconnected and converge on mTOR signaling to control tumor cell growth [34]. A previous study shows that zebrafish Kras-induced liver tumors had simultaneous activation of the Ras and PI3K-AKT pathways [35]. Our immunohistochemical analyses revealed no activation of the PI3K-AKT pathway, but showed increased expression of p4E-BP1 and pS6RP, two main targets of the mTOR pathway. There is the possibility that these two targets were activated by Ras itself, as RAS has been shown to be able to activate mTOR downstream targets through both mTOR-dependent and independent mechanisms [36].

In mouse models of Kras-induced glioma, expression of oncogenic Ras is often insufficient for malignant gliomagenesis [6]. Our current study demonstrated that oncogenic KRAS itself was sufficient to initiate gliomagenesis in transient transgenic situations, albeit with rather long latencies. Surprisingly, a previous study using the zebrafish *nestin* promoter to drive conditional expression of the zebrafish version of Kras^G12V^ did not result in brain tumors [37]. Our current study also showed that *gfap*-driven expression of oncogenic KRAS in a stable line did not lead to increased mitosis during early brain development. These findings raise the issue that other oncogenic events may be needed for efficient brain tumorigenesis in zebrafish. Since the constitutively active form of AKT is required for mouse gliomagenesis [6,20], it will be interesting to test whether brain tumor penetrance and malignancy could be enhanced by co-activation of the PI3K-AKT pathway.

### Zebrafish tumor models for drug screening

A distinct advantage of zebrafish cancer models lies in their ability to be used for screening small molecules to identify drugs of therapeutic value in *in vivo* settings and in a relatively high-throughput manner [38]. Recent drug screening efforts in zebrafish have uncovered small molecules that can potentially be used to treat human melanoma [39] and leukemia [40]. The *krt5*-derived transgenic line with the early skin hyperplasia phenotype could be explored as an *in vivo* platform to screen for oncogenic KRAS inhibitors [41]. As for the *gfap*-derived transgenic line, larvae as early as 72 hpf had significantly increased mitotic activity, as indicated by a dramatic increase of pH3-positive cell numbers in the spinal cord (Figure 6). Since pH3 immunostaining has been successfully used as a marker for high-throughput chemical screenings in zebrafish [42], we believe our transgenic line could be further evaluated for screening small molecules that inhibit the mitotic effects of oncogenic KRAS in the CNS. Drug leads from these screening can then be used for identifying candidates that penetrate into the brain, as zebrafish also possess a functional BBB expressing multidrug resistance proteins [43,44].

## Conclusions

Our study showed that oncogenic KRAS promoted brain tumors in zebrafish, and that tumorigenesis was driven by the activation of the canonical Ras and mTOR pathways. Zebrafish provide an invaluable model for understanding brain tumor cell origin, mechanisms of brain tumorigenesis, and may serve as *in vivo* platforms for studying cancer gene functions and for screening drugs to inhibit the oncogenic effects of RAS mutations.

## Methods

### Zebrafish husbandry

The AB strain was purchased from the Zebrafish International Resource Center (ZIRC, Eugene, OR). Embryos and larvae were maintained at 28.5°C in egg water (0.03% Instant Ocean). All experiments on transgenic expression of oncogenes and handling of transgenic fish with tumors were approved by the St. Jude Children’s Research Hospital Institutional Animal Care and Use Committee.

### Plasmid DNA construction and transgenesis

The Gateway system was adapted to generate promoter-containing driver constructs [45]. The *krt5* and *gfap* promoter sequences were inserted into p5E and the *Gal4VP16* sequence was inserted into pME. The p5E vectors containing the respective promoters and the pME-Gal4VP16 were combined with the 3’ entry clone *p3E-polyA* and the destination vector *pDestTol2pA2* to create the construct *krt5:Gal4VP16, gfap:Gal4VP16* using the LR Clonase II Plus Enzyme mix (Invitrogen, Carlbad, CA). The *pIUI-mCherry-KRAS*^*G12V*^ effector construct was made with the I-SceI meganuclease system as previously described [21]. About 20 pg of combined driver and effector plasmid DNA together with about 30 pg of Tol2 transposase mRNA and 0.001 unit of I-SceI meganuclease (New England BioLabs) were injected into 1-cell stage eggs in 1–2 nl volume.

To generated stable transgenic lines, we adopted the Clontech Tet-On® inducible system (Clontech, Montain View, CA). Briefly, the Tet-On fragment of the pTet-On® advanced vector (CAT No. 631069) was cloned into the pME of the gateway system to generate the *krt5:Tet-On*^*AD*^ and *gfap: Tet-On*^*AD*^ driver constructs. The TRE-Tight fragment from the pTRE-Tight vector (CAT No. 631059) and the mCherryKRAS^G12V^ oncogene were directly cloned into the pT2AL200R150G vector [46] to generate the TRE:mCherryKRAS^G12V^ effector construct. The driver and effector constructs were co-injected into 1-cell-stage eggs. Stable transgenic fish were selected from F1 embryos showing mCherry expression after treatment with 10 μg/ml doxycycline (Clontech, Mountain View, CA).

### Brain tumor pathology and immunohistochemistry

Zebrafish harboring tumors were euthanized using 0.04% Tricaine and fixed in 4% paraformaldehyde for 2 days. Fish were then decalcified using 0.5 M EDTA (AMRESCO®) for 5 days, rinsed in phosphate buffered saline, dehydrated, and paraffin wax embedded. 5 μm sections were stained using hematoxylin and eosin (H&E). Immunofluorescence on paraffin sections and cryosections were conducted using the Cell Signaling Technology protocol that accompanies the purchased antibodies. The primary anti-rabbit antibodies used are: GFAP (Dako, Z0334, 1:1000), S100 (Dako, Z0311, 1:2000), pERK1/2 (Cell Signaling, 4370, 1:200), pAKT (Ser473) (Cell Signaling, 4060, 1:200), pS6RP (Cell Signaling, 2211, 1:200), p4E-BP1 (Cell Signaling, 2855P, 1:200), and pH3(Ser10) (Cell Signaling, 9701, 1:50). The secondary antibodies used are Alexa Fluor®488 (A-11034) and Alexa Fluor®568 (A-11011) anti-Rabbit IgG (H + L) (Life Technologies, 1:2000).

### Doxycycline and U0126 treatment of embryos

Doxycycline (Clontech, Mountain View, CA) was dissolved in distilled water at the stock concentration of 10 mg/ml. Doxycycline at the final concentration of 10 μg/ml (unless stated otherwise) were added to embryos at the early gastrula stage to induce transgenic expression. The MEK inhibitor U0126 (LC labs, Woburn, MA) was dissolved in DMSO at a stock concentration of 100 mM. Early gastrula-stage embryos were either treated with Doxycycline alone to induce transgenic expression or treated with the combination of Dox and 50–100 μM U0126 to show oncogenic KRAS inhibition.

## Additional file

Additional file 1: Figure S1. Tg*(krt5-EGFP)* expression during development. (A) Lateral view of a 72 hpf larva showing EGFP expression in skin epithelial cells. (B) A72 hpf larva showing sporadic EGFP expression in brain (arrows). (C) A 3-week-old juvenile showing EGFP expression in brain radial glial cells (arrow) and chondrocytes (arrowhead). OT, optic tectum; DI, diencephalon. Scale bars, 1 mm, A; 100 μm, B and C. **Figure S2.** Antibody cross-reactivity in tumor paraffin-sections. (A) A *krt5*-derived tumor showing GFAP reactivity at the ventricular zone (arrow), not tumor mass. (B) The same tumor showing S100β reactivity at the ventricular zone (arrow), not tumor mass. (C) A tumor from coexpression of oncogenic smoothened and AKT1 showing expression of pAKT(S473). (D) A *gfap*-derived tumor was negative for pAKT(S473). OT, optic tectum. Scale bars, 40μm. **Figure S3.** Expression of *krt5:KRASG12V* resulted in MPNST-like tumors. (A) A 6-month-old fish showing a tumor in anterior trunk. (B) Tumor cells exhibits spindle, epithelioid cell morphologies with mitotic figures (arrows). (C) An 8-month-old tumor obliterated the ventral brain, invaded the gills. (D) Enlarged view of tumor in (C) showing spindle, epithelioid cells and mitotic figure (arrow). Scale bars, 200 μm, A, C; 20 μm, B, D. **Figure S4.** Expression of *gfap:KRASG12V* resulted in undifferentiated neoplasms. (A) A 12-month-old fish showing a large tumor mass in the anterior trunk. (B) Tumor cells exhibits spindle, epithelioid cell morphologies. (C) A 4-month-old tumor infiltrated the lower jaw. (D) Enlarged view of (C) showing compact and round tumor cells. Scale bars, 200μm, A, C; 20 μm, B, D. **Figure S5.** KRAS inhibition in stable transgenic fish. (A, B) 48 hpf Tg(*Krt5:rtTA:mCherryKRASG12V)* transgenic larvae showing skin hyperplasia (arrows), which was eliminated by 50μM U0126 treatment. (C, D) In Tg(*gfap:rtTA:mCherryKRASG12V)* stable line, 100μM U0126 treatment reduced KRAS expression in CNS, but caused developmental defects.

## Abbreviations

CNS: Central nervous system
BBB: Blood brain barrier
DIPG: Diffuse intrinsic pontine glioma
GBM: Glioblastoma multiforme
Krt5: Cytokeratin 5
GFAP: Glial fibrillary acidic protein
EGFP: Enhanced green fluorescent protein
OT: Optic tectum
VZ: Ventricular zone
MPNST: Malignant peripheral nerve sheath tumor
PNET: Primitive neuroectodermal tumor
H&E: Hematoxylin and eosin
MEK: MAP kinase kinase
mTOR: Mammalian target of rapamycin
PI3K: Phosphatidylinositide 3-kinases
4E-BP: 4E-binding protein
rpS6: Ribosome protein S6
pH3: Phosphorylated Histone 3
UAS: Upstream activating sequence
Dox: Doxycycline
rTetR: Reverse tetracycline repressor
TRE: Tetracycline-response elements
