# Supplementary material for: Oncogenic KRAS promotes malignant brain tumors in zebrafish
Source: Mol Cancer. 2015 Feb 3;14(1):18. doi: 10.1186/s12943-015-0288-2 (PMC4320811; doi:10.1186/s12943-015-0288-2)
Supplement: Additional file 1: Figure S1. — Tg(krt5-EGFP) expression during development. (A) Lateral view of a 72 hpf larva showing EGFP expression in skin epithelial cells. (B) A72 hpf larva showing sporadic EGFP expression in brain (arrows). (C) A 3-week-old juvenile showing EGFP expression in brain radial glial cells (arrow) and chondrocytes (arrowhead). OT, optic tectum; DI, diencephalon. Scale bars, 1 mm, A; 100 μm, B and C. Figure S2. Antibody cross-reactivity in tumor paraffin-sections. (A) A krt5-derived tumor showing GFAP reactivity at the ventricular zone (arrow), not tumor mass. (B) The same tumor showing S100β reactivity at the ventricular zone (arrow), not tumor mass. (C) A tumor from coexpression of oncogenic smoothened and AKT1 showing expression of pAKT(S473). (D) A gfap-derived tumor was negative for pAKT(S473). OT, optic tectum. Scale bars, 40μm. Figure S3. Expression of krt5:KRASG12V resulted in MPNST-like tumors. (A) A 6-month-old fish showing a tumor in anterior trunk. (B) Tumor cells exhibits spindle, epithelioid cell morphologies with mitotic figures (arrows). (C) An 8-month-old tumor obliterated the ventral brain, invaded the gills. (D) Enlarged view of tumor in (C) showing spindle, epithelioid cells and mitotic figure (arrow). Scale bars, 200 μm, A, C; 20 μm, B, D. Figure S4. Expression of gfap:KRASG12V resulted in undifferentiated neoplasms. (A) A 12-month-old fish showing a large tumor mass in the anterior trunk. (B) Tumor cells exhibits spindle, epithelioid cell morphologies. (C) A 4-month-old tumor infiltrated the lower jaw. (D) Enlarged view of (C) showing compact and round tumor cells. Scale bars, 200μm, A, C; 20 μm, B, D. Figure S5. KRAS inhibition in stable transgenic fish. (A, B) 48 hpf Tg(Krt5:rtTA:mCherryKRASG12V) transgenic larvae showing skin hyperplasia (arrows), which was eliminated by 50μM U0126 treatment. (C, D) In Tg(gfap:rtTA:mCherryKRASG12V) stable line, 100μM U0126 treatment reduced KRAS expression in CNS, but caused developmental defects. [file 12943_2015_288_MOESM1_ESM.pdf]

Additional files

Additional file as PDF.

Figure S1. *Tg(krt5-EGFP)* expression patterns during early stages of fish development. (**A**) Lateral view (anterior to the left) of a transgenic larva at 72 hpf showing EGFP expression predominantly in skin epithelial cells. (**B**) Transverse section of a 72 hpf larvae showing sporadic EGFP expression in brain (arrows). (**C**) Transverse section through the hindbrain of a 3-week old transgenic juvenile showing EGFP expression in radial glial cells of ventricular zones (arrow) and chondrocytes (arrowhead). OT, optic tectum; DI, diencephalon. Scale bars, 1 mm for A; 100 μm for B and C.

Figure S2. Validation of antibody cross-reactivity in paraffin-sections of zebrafish tumor samples. (**A**) A *krt5*-derived brain tumor showed GFAP reactivity at the brain ventricular zone (arrow), but not in the tumor mass. (**B**) The same sample showed S100β reactivity at the ventricular zone (arrow), but not in the tumor mass. (**C**) A tumor sample derived from co-expression of zebrafish oncogenic smoothened and human constitutively active AKT1 showed prominent expression of pAKT(S473. (**D**) A *gfap*-derived brain tumor was negative for pAKT(S473). The weak fluorescence observed in OT is non-specific staining from the secondary antibody. OT, optic tectum. Scale bars, 40μm.

Figure S3. Transient expression of *krt5:KRAS*^{G12V} resulted in MPNST-like tumors. (**A**) H&E staining of sagittal section from a 6-month-old fish showing a large tumor mass in the anterior trunk region. (**B**) Tumor cells exhibited spindle and epithelioid cell morphologies.

Mitotic figures were prevalent within the tumor mass (arrows). (**C**) A8-month-old fish with tumor cells obliterating the ventral brain and invading the gills. (**D**) Enlarged view of tumor cells in (C) showing spindle and epithelioid cells and a mitotic figure (arrow). Scale bars, 200 μm for A, C; 20 μm for B, D.

Figure S4. Transient expression of *gfap:KRAS*^{G12V} resulted in undifferentiated neoplasms. (**A**) H&E staining of sagittal section from a 12-month old fish showing a large tumor mass in the anterior trunk region. (**B**) Tumor cells exhibited spindle and epithelioid cell morphologies. (**C**) A 4-month-old fish with tumor cells infiltrating the lower jaw. (**D**) Enlarged view of (C) showing compact and round tumor cells. Scale bars, 200μm for A, C; 20 μm for B, D.

Figure S5. Inhibition of oncogenic KRAS expression in stable transgenic fish. (**A, B**) Tg(*Krt5:rtTA:mCherryKRAS*^{G12V}*)* transgenic larvae showed skin hyperplasia at 48hpf (arrows) which could be eliminated by 50μM U0126 applied at early gastrula stage. (**C, D**) In a Tg(*gfap:rtTA:mCherryKRAS*^{G12V}*)* stable line, 100μM U0126 treatment could significantly reduce oncogenic KRAS expression in CNS at 48hpf, but caused severe developmental defects.

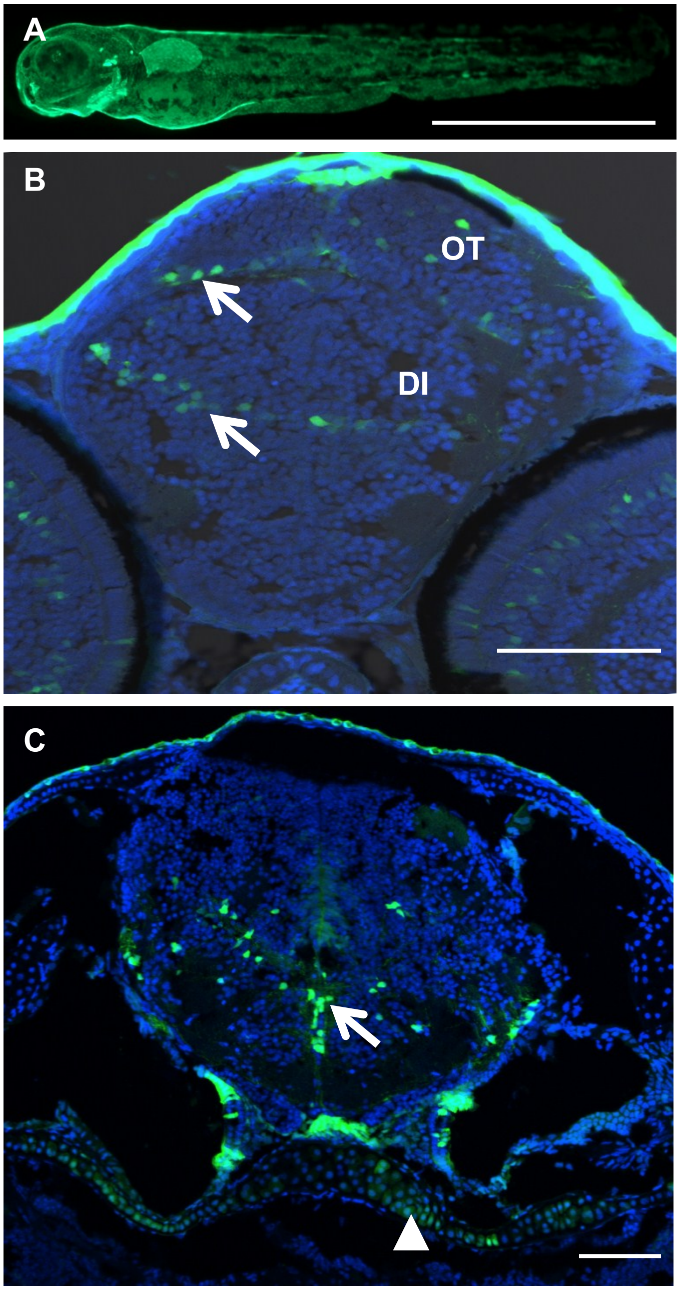

Figure S1

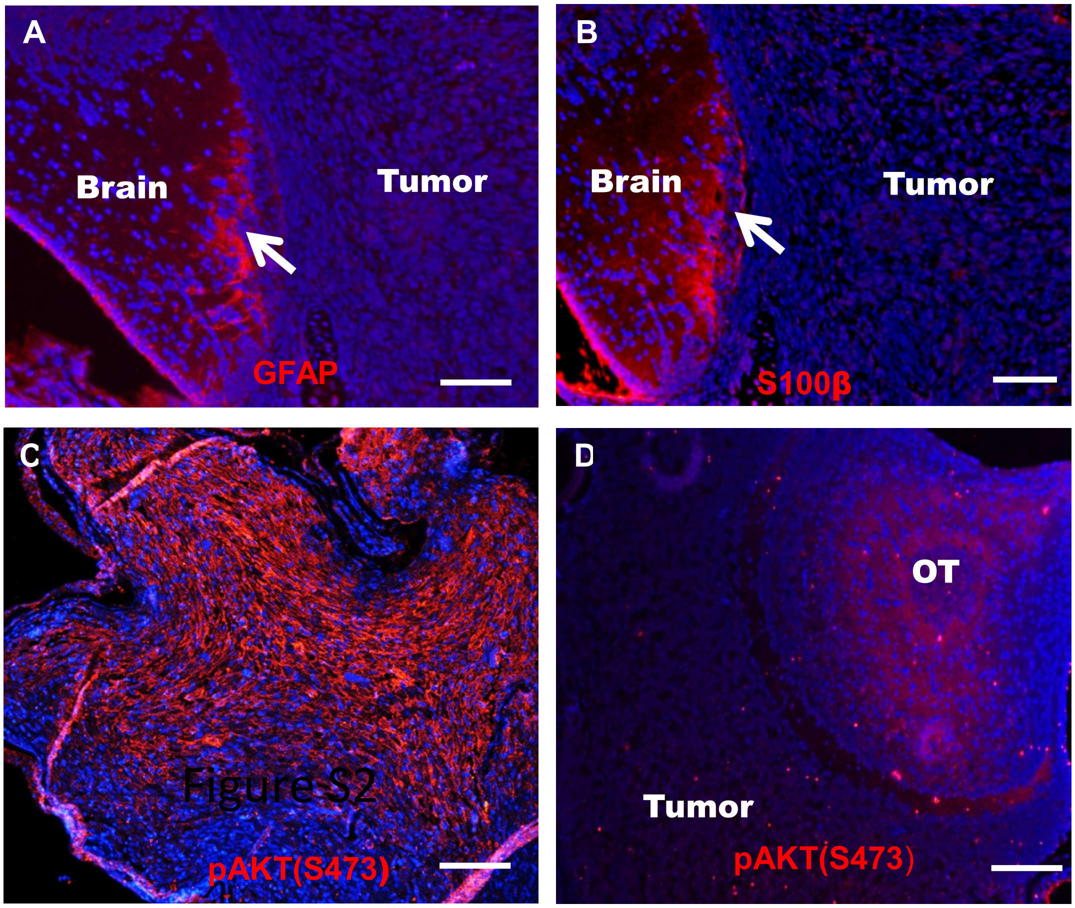
A
Brain
Tumor
GFAP
B
Brain
Tumor
S100β
C
Figure S2
pAKT(S473)
D
OT
Tumor
pAKT(S473)

Figure S2

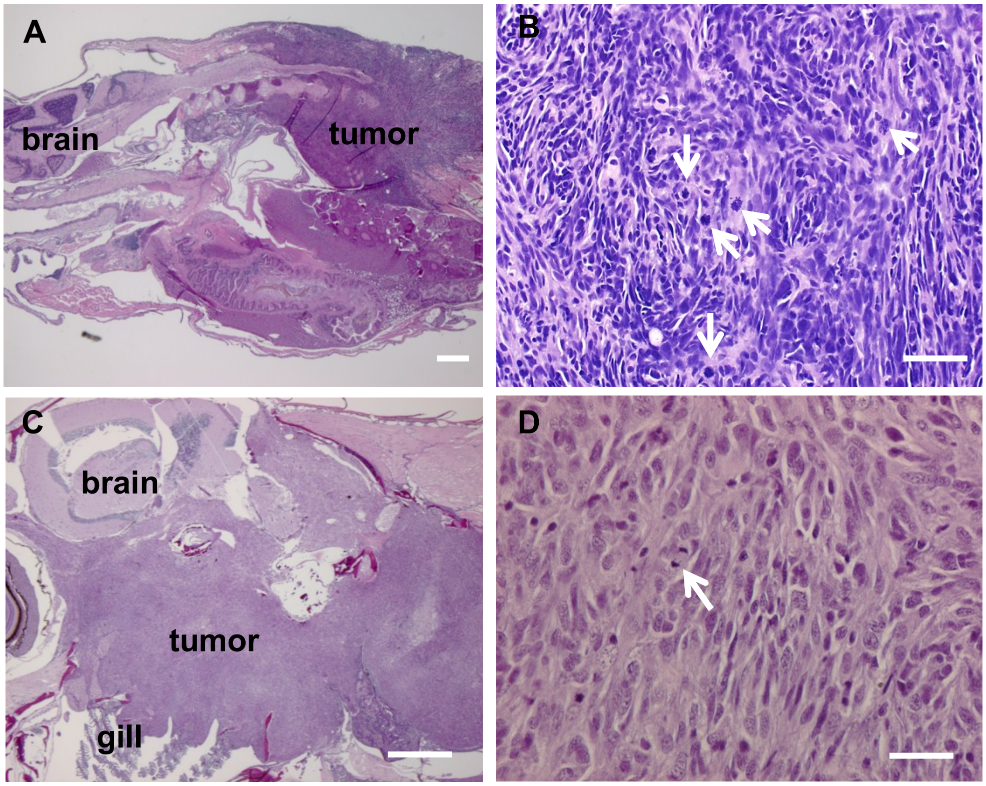

Figure S3

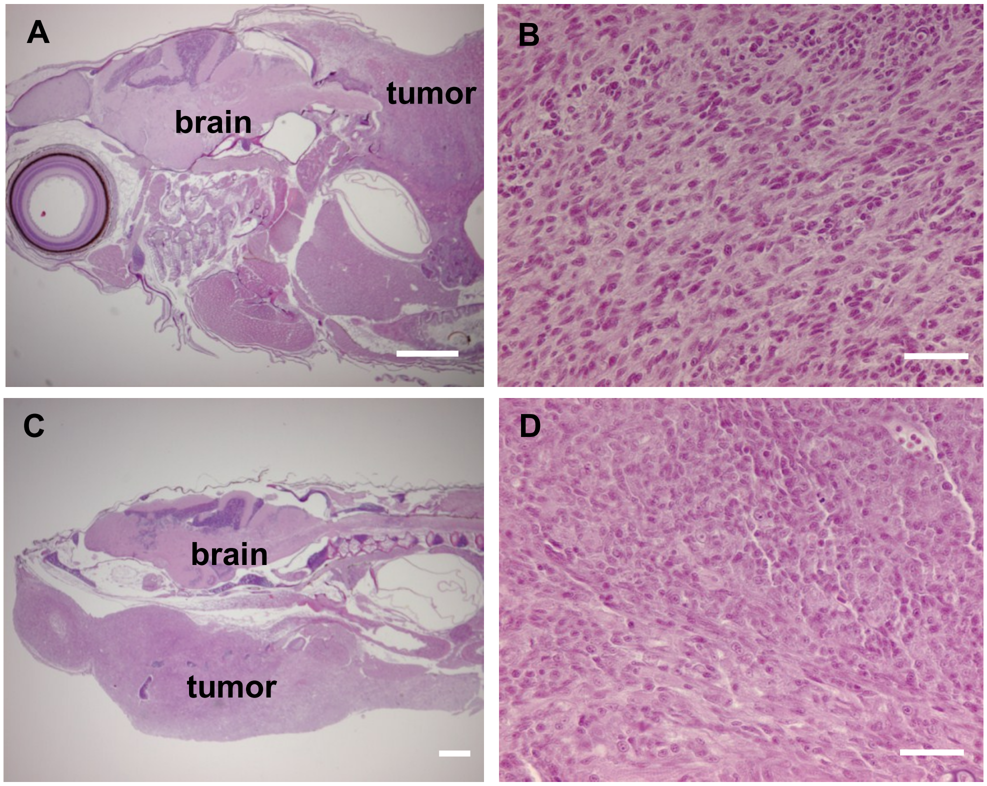

Figure S4

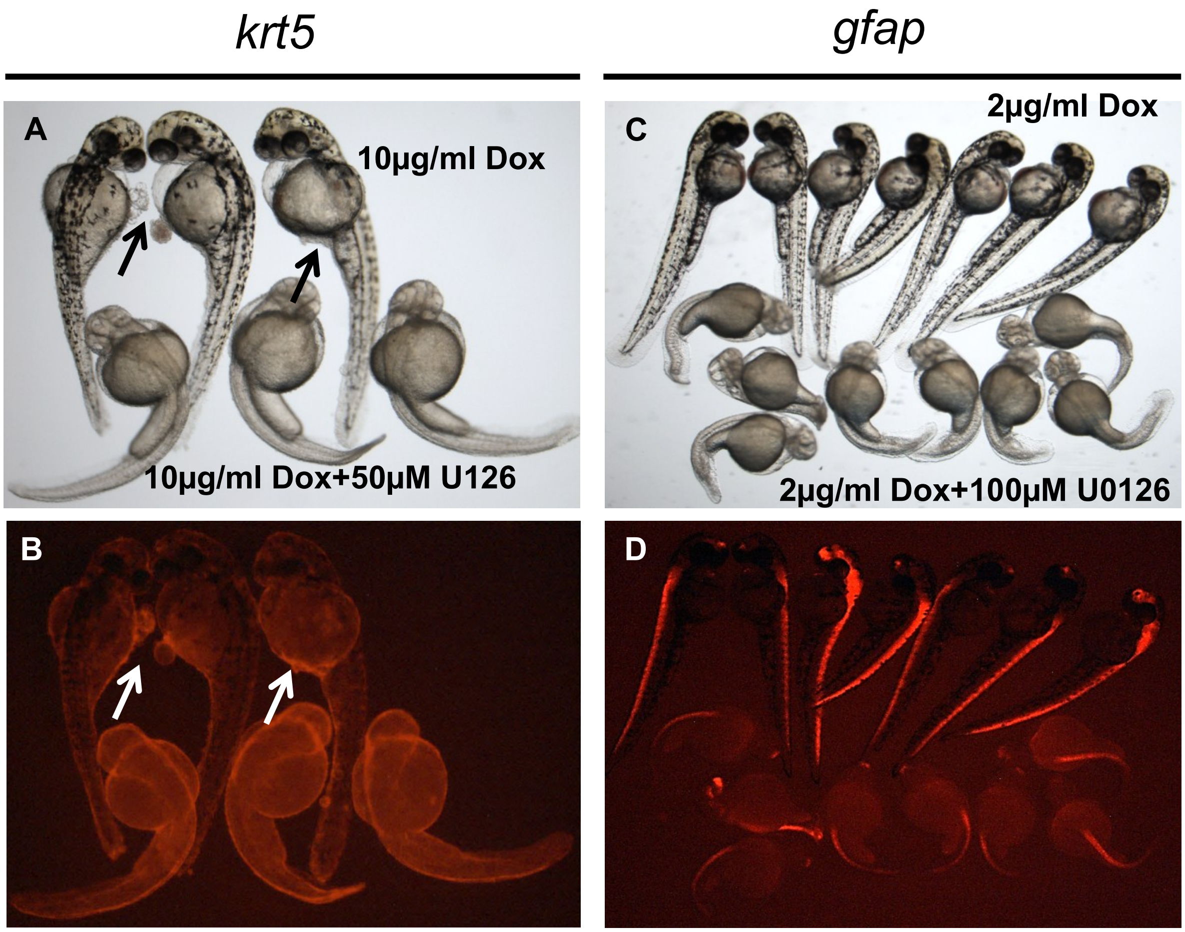
krt5
gfap
A
10µg/ml Dox
10µg/ml Dox+50µM U126
C
2µg/ml Dox
2µg/ml Dox+100µM U0126
B
D

Figure S5
